# Activation of Thromboxane A_2_ Receptor (TP) Increases the Expression of Monocyte Chemoattractant Protein -1 (MCP-1)/Chemokine (C-C motif) Ligand 2 (CCL2) and Recruits Macrophages to Promote Invasion of Lung Cancer Cells

**DOI:** 10.1371/journal.pone.0054073

**Published:** 2013-01-17

**Authors:** Xiuling Li, Hsin-Hsiung Tai

**Affiliations:** Department of Pharmaceutical Sciences, College of Pharmacy, University of Kentucky, Lexington, Kentucky, United States of America; Sun Yat-sen University Medical School, China

## Abstract

Thromboxane synthase (TXAS) and thromboxane A_2_ receptor (TP), two critical components for thromboxane A_2_ (TXA_2_) signaling, have been suggested to be involved in cancer invasion and metastasis. However, the mechanisms by which TXA_2_ promotes these processes are still unclear. Here we show that TXA_2_ mimetic, I-BOP, induced monocyte chemoattractant protein -1(MCP-1)/chemokine (C-C motif) ligand 2 (CCL2) expression at both mRNA and protein levels in human lung adenocarcinoma A549 cells stably over-expressing TP receptor α isoform (A549-TPα). The induction of MCP-1 was also found in other lung cancer cells H157 and H460 that express relatively high levels of endogenous TP. Using specific inhibitors of several signaling molecules and promoter/luciferase assay, we identified that transcription factor SP1 mediates I-BOP-induced MCP-1 expression. Furthermore, supernatants from I-BOP-treated A549-TPα cells enhanced MCP-1-dependent migration of RAW 264.7 macrophages. Moreover, co-culture of A549 cells with RAW 264.7 macrophages induced expression of *MMPs*, *VEGF* and *MCP-1* genes, and increased the invasive potential in A549 cells. These findings suggest that TXA_2_ may stimulate invasion of cancer cells through MCP-1-mediated macrophage recruitment.

## Introduction

There is overwhelming evidence showing that chemokines play a significant role in tumor progression, especially in cancer invasion and metastasis [1]. Chemokines are a superfamily of biologically active peptides which comprises about 50 members so far. Among these many chemokines monocyte chemoattractant protein -1/chemokine (C-C motif) ligand 2 (MCP-1/CCL2), a member of the CC chemokines subfamily, is of particular relevance to cancer invasion and metastasis. MCP-1 is over-expressed in a variety of cancer types including glioma, ovarian, lung, breast and prostate cancer [2]–[4]. As its name indicates, MCP-1 is a potent chemoattractant for monocytes/macrophages. It has been demonstrated that MCP-1-mediated macrophage infiltration promotes tumor progression in various types of cancer. For example, MCP-1 secreted by breast tumor cells recruits inflammatory monocytes that produce VEGF to promote tumor cells extravasation and lung metastasis [5], [6]. MCP-1 also enhances prostate tumor growth and angiogenesis by recruitment of monocytes and tumor associated macrophages (TAMs) into the tumor microenvironment [7]. In addition, tumor cells derived from renal cancer recruit tumor-infiltrating lymphocytes (TIL) by secreting MCP-1 and IL-8 which contribute to renal cancer progression [8]. Moreover, CC chemokines including MCP-1 and MIP-1β were found to be associated with macrophage infiltration in human non-small cell lung cancer (NSCLC) tumors. Patients with recurrence of the disease were found to have higher macrophage infiltration in their initial tumors [9]. In addition to tumor cells themselves, tumor associated stromal cells such as endothelial cells, fibroblasts and macrophages also produce a significant amount of MCP-1 to increase TAM infiltration and maintain inflammation, therefore, promoting tumor progression [10], [11].

MCP-1 can be induced by a variety of growth factors and cytokines, such as platelet-derived growth factor (PDGF) [12], tumor necrosis factor alpha (TNFα) [13], interferon gamma (IFN-γ) [14], and IL-1β [15]. Recent studies have found that arachidonate metabolites, prostaglandin E_2_ (PGE_2_) and 15(*S*)-HETE also induce the expression of MCP-1 [16], [17]. Blockade of thromboxane A_2_ (TXA_2_) receptor (TP) suppressing the expression of MCP-1 in human umbilical vein endothelial cells has been reported as well [18]. However, whether activation of TP can induce MCP-1 expression and release from cancer cells remains to be determined.

TXA_2_ is generated from prostaglandin H_2_ (PGH_2_) by the action of thromboxane synthase (TXAS) [19]. Levels of TXAS have been reported to correlate with the invasion and metastasis potential of cancer cells [20]–. TXA_2_ mimetic, U46619, was also found to stimulate the invasion of bladder cancer cells [23]. Nonetheless, the critical molecules that mediate TXA_2_-stimulated invasion and progression of cancer cells need to be identified.

We recently reported that TP agonist, I-BOP, induced A549-TPα cells invasion via induction of several MMPs [24]. In the current study, we show that I-BOP also induced the expression of MCP-1 in A549-TPα cells which exhibited a chemotactic effect on RAW 264.7 macrophages. Co-culture of A549 cells or A549-TPα cells with RAW 264.7 macrophages also increased the expression of *MMPs*, *VEGF* and *MCP-1* genes, and the invasion by A549 cells. These findings suggest that MCP-1 is an important mediator for TXA_2_-stimulated invasion of cancer cells.

## Materials and Methods

### Reagents

I-BOP, SQ29548, PGD_2_, PGE_2_, PGF_2α_ were from Cayman Chemical (Ann Arbor, MI). GF109203X was from Calbiochem (San Diego, CA). U0126 was from Alexis Biochemicals (San Diego, CA). RS-102895, mithramycin A, geldanamycin and other biochemicals and chemicals were from Sigma-Aldrich (St. Louis, MO). Recombinant human MCP-1 and polyclonal antibody directed against MCP-1 and characterized for neutralizing activity were from PeproTech (Rocky Hill, NJ). Antibodies against SP1 and E-cadherin were purchased from Santa Cruz Biotechnology (Santa Cruz, CA). Antibody against glyceraldehyde-3-phosphate dehydrogenase (GAPDH) was generated in house as previously described [25].

### Cell Lines and Cell Culture

Human lung adenocarcinoma cell line A549 overexpressed TPα or TPβ and the control A549 cells overexpressed GFP were established and identified as previously described [24]. These cells were designated as A549-TPα, A549-TPβ and the control A549 cells accordingly. All these cells and other two lung cancer cell lines H157 and H460 were maintained in RPMI1640 medium supplement with 10% heat inactivated fetal bovine serum (FBS), 0.1 mg/mL streptomycin and 100 U/mL of penicillin G (Invitrogen, Carlsbad, CA) at 37°C in a humidified atmosphere of 95% air and 5% CO_2_. Mouse Raw 264.7 macrophages were maintained in Dulbecco’s modified Eagle’s medium (DMEM). All cells were originally obtained from the American Type Culture Collection (ATCC; Manassas, VA).

### Cell Treatment and Western Blot

Cells were plated in 12-well plates to achieve ∼80% confluence next day, and then were starved in RPMI 1640 medium without FBS for 24 h before stimulation. For inhibition study, cells were pretreated with the respective inhibitors at working concentrations for 30 min in serum-free medium prior to stimulation. After a certain time of treatment, cells or cultured media were collected for Western blot analysis as described previously [26]. Intensities of Western blot bands were quantified using NIH Image J software.

### MCP-1 Precipitation

MCP-1 in cell culture media was precipitated by using trichloroacetic acid (TCA)-acetone method. Briefly, TCA was added to media at a final concentration of 10%. Proteins were precipitated for 4 h on ice. Precipitated proteins were centrifuged at 5,000×*g* for 30 min, washed three times in cold acetone, and air dried. Total protein was quantified by the Bio-Rad protein assay (Bio-Rad Laboratories, Inc., Hercules, CA) to ensure equal loading of proteins for Western blot analysis.

### Reverse Transcription-polymerase Chain Reaction (RT-PCR)

Total RNA was isolated from cells using the TRI Reagent (Sigma-Aldrich, St. Louis, MO) and reverse transcribed using the SuperScript II reverse transcriptase (Invitrogen, Calsbard, CA). The PCR primers for MCP-1 gene amplification were 5′-ATAGCAGCCACCTTCATTCC-3′ (forward) and 5′-TTCCCCAAGTCTCTGTATCT-3′(reverse); for MMP-1 were 5′-CATTGATGGCATCCAAGC-3′(forward) and 5′-CCGGACTTCATCTCTGT-3′(reverse); for MMP-9 were 5′-GGCGCTCATGTACCCTATGT-3′(forward) and 5′-TCAAAGACCGAGTCCAGCTT-3′ (reverse); for MMP-10 were 5′-GTCACTTCAGCTCCTTTCCT-3′(forward) and 5′-ATCTTGCGAAAGGCGGAACT-3′(reverse); for MMP-13 were 5′-AGTGGTAAGAATAGTAGATGTG-3′(forward) and 5′-GGCCGATCATATATTCAATAAGT-3′(reverse); for VEGF 5′-GGATGTCTATCAGCGCAGCTAC-3′(forward) and 5′-TCACCGCCTCGGCTTGTCACATC-3′(reverse); for β-actin 5′-GGCATGGGTCAGAAGGATTCC-3′(forward) and 5′-AGCACAGCCTGGATAGCAACG-3′(reverse). PCR conditions were 2 min at 95°C followed by 35 cycles (23 cycles for MCP-1 and β-actin) of 95°C for 1 min, 50–60°C for 30 s and 72°C for 1 min. The PCR product of each sample was analyzed by electrophoresis in a 1.5% agarose gel and visualized by ethidium bromide staining.

### Real-time PCR Analysis of MCP-1 mRNA

The real-time PCR was performed with human MCP-1 and β-actin (as an internal standard)-specific primers. The sequence of the MCP-1 forward primer was 5′-ATAGCAGCCACCTTCATTCC-3′, and the sequence of MCP-1 reverse primer was 5′-ATCCTGAACCCACTTCTGCT-3′. The sequence of β-actin forward primer was 5′-AGAAAATCTGGCACCACACC-3′, and the sequence of β-actin reverse primer was 5′-AGAGGCGTACAGGGATAGCA-3′. All real-time PCR reactions were carried out in a final volume of 50 µl and were performed in duplicate for each cDNA sample in the ABI PRISM 7700 Sequence Detection System according to the manufacturer’s protocol. The optimized reaction consisted of 25 µl of iTaq SYBRGreen Supermix with ROX, 0.02 U/µl of Uracil-N-glycosylase, 3 µl of diluted cDNA templates, and 200 nM of each specific forward and reverse primer. The PCR protocol was 95°C for 5 min, followed by 45 cycles of 95°C for 15 s and 60°C for 1 min. Specificity of the amplification was checked by melt-curve analysis. Relative levels of mRNA expression were calculated according to Pfaffl method [27]. Individual expression values were normalized by comparison with β-actin mRNA expression.

### Promoter Construct and Site-directed Mutagenesis

Human MCP-1-CAT reporter plasmid was kindly provided by Dr. Bassel E. Sawaya (Temple University, Philadelphia, PA) and reconstructed into pGL3 luciferase reporter vector. A site-directed mutagenesis kit (Stratagene, La Jolla, CA) was used to mutate the core sequence of SP1 binding site in the reconstructed luciferase reporter plasmid; SP1 wild-type (SP1wt), 5′-CCGCCC-3′ and SP1 mutant (SP1m), 5′-CCGggg-3′.

### Luciferase Assay

Cells were seeded in 12-well plates and transfected with pGL3-MCP-1 reporter plasmids using TransIT-2020 Transfection Reagent (Mirus Bio LLC, Madison, WI). After 24 h of transfection, cells were incubated with I-BOP or control (0.1% ethanol) for additional 18 h. Thereafter, cells were collected and further detected by using a microplate luminometer (MTX lab systems, Vienna, VA). The luciferase activity in cell lysate was determined as described previously [25].

### Preparation of Nuclear Extract

Nuclear proteins were prepared as described previously [28]. In brief, A549-TPα cells were scraped into cold phosphate buffered saline (PBS) and centrifuged at 1000 rpm for 2 min. Pellets were resuspended in buffer A [10 mM HEPES-KOH pH 7.9, 1.5 mM MgCl_2_, 10 mM KCl, 0.5 mM dithiothreitol (DTT) and 0.2 mM phenylmethylsulfonyl fluoride (PMSF)] and incubated on ice for 15 min, then centrifuged at full speed in a tabletop centrifuge. The nuclear pellets were lysed for 20 min on ice in buffer B (20 mM HEPES-KOH, 1.5 mM MgCl_2_, 420 mM NaCl, 25% glycerol, 0.2 mM EDTA, 0.5 mM DTT and 0.2 mM PMSF), and centrifuged for 2 min as above. The supernatant containing nuclear proteins was frozen at −80°C until analyzed.

### Preparation of Conditioned Medium

A549-TPα cells were plated into 10 cm dishes to achieve ∼80% confluence next day, then media were switched to 10 ml serum free RPMI1640. After 24 h of starvation, cells were treated with vehicle (0.1% ethanol) or I-BOP (50 nM) in fresh serum free medium for another 24 h. Then, conditioned medium was collected and passed through 30 kDa and 10 kDa cutoff centrifugal filters (Millipore, Billerica, MA) sequentially (MCP-1 molecular weight is 13–15 kDa). The remains on 10 kDa filter were dissolved in 1.5 ml serum free medium and stored at −80°C until used.

### Macrophage Migration Assay

RAW 264.7 macrophages migration was assessed by using CytoSelect 96-well cell migration assay kit (Cell Biolabs, San Diego, CA) following the manufacturers instruction. In brief, ∼1.0×10^5^ cells in serum free medium were seeded onto the top well of a Trans-well insert (8 µm polycarbonate nucleopore filters), and the bottom well was supplemented with 150 µl serum free medium or A549-TPα conditioned medium prepared above. After 12 h of stimulation, cells that had migrated through the filter were detached and dyed with CyQuant GR dye solution. The fluorescence was measured at 480/520 nm. For antibody neutralization study, 150 µl serum free medium or conditioned medium was incubated with MCP-1 neutralizing antibody (5 µg) or isotype control antibody (5 µg) for 2 h at 37°C before added to the lower chamber. For inhibition study, RAW 264.7 cells were incubated with MCP-1 receptor (CCR2) antagonist RS-102895 (10 µM) for 30 min before stimulation with the conditioned medium.

### Co-culture Experiments

For transwell co-culture system, control A549 or A549-TPα cells were plated into a six-well culture plate, and RAW 264.7 cells were seeded onto transwell inserts (with a 0.4 µm pore size) top on another six-well plate. After 24 h, the media of A549 and RAW cells were replaced with fresh serum free media and the RAW 264.7 transwell inserts were moved onto the six-well plate where A549 cells were seeded. After 12 h of co-culture, A549 cells were pictured and collected for RT-PCR analysis. For direct co-culture, control A549 cells which express GFP were plated with or without RAW 264.7 cells into a 12-well plate and cells were pictured after a certain periods. The images of cells were captured using a Kodak digital camera under Olympus Tokyo CK inverted microscope.

### Invasion Assay

The invasion assay was carried out following the instructions of Cultrex 24-well Transwell BME cell invasion assay (Trevigen). Briefly, a 24-well unit with 8 µm polycarbonate nucleopore filters (Corning) evenly coated with 100 µl basement membrane extract coating solution (Trevigen) at 37°C for 4 h. Control A549 cells (2×10^5^) expressing GFP and RAW 264.7 macrophages (1×10^5^) in serum free medium were placed in the upper compartment, and 50% RPMI 1640 plus 50% DMEM medium supplied with 0.5% FBS was added to the lower compartment. After 24 h incubation, cells that had not invaded were removed with a cotton swab. Cells that had invaded the lower surface of the membrane were observed under a fluorescence microscope. Invaded cells in three randomly selected fields were counted.

### Statistical Analysis

The differences between each group were expressed as mean ± SD. Statistical significance was assessed by Student’s *t* test. Differences were considered statistically significant when *P* values were ≤0.05.

## Results

### Induction of MCP-1 Expression by Activation of TP

**Figure 1:**
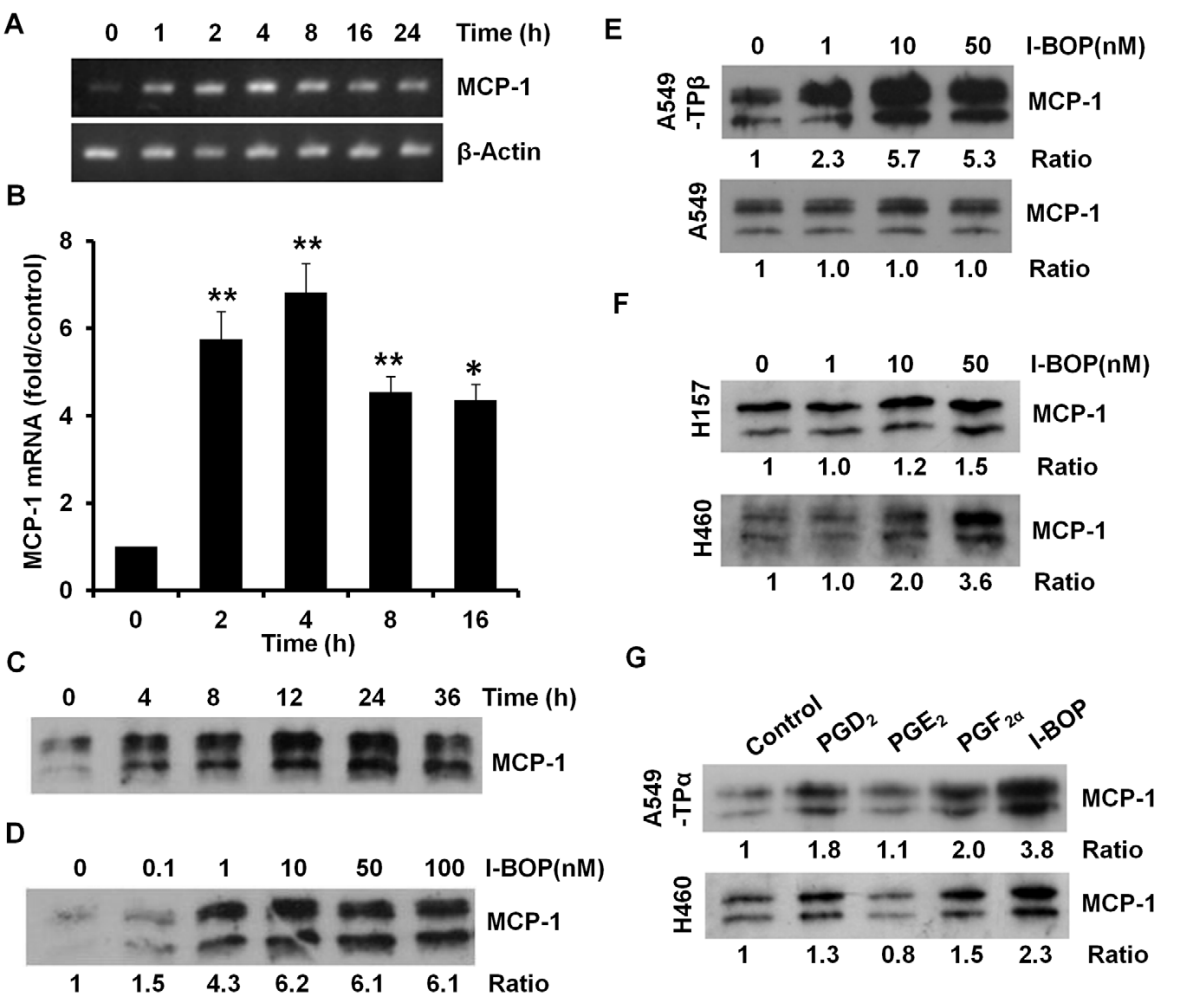
Effects of I-BOP on MCP-1 expression. **A** I-BOP induced transcription of MCP-1. Cells were serum-starved for 24 h before treated with 50 nM I-BOP for the indicated time periods. RNAs were isolated and RT-PCR was carried out as described in Materials and methods. **B** MCP-1 mRNA induction in A was confirmed by quantitative real-time PCR. **p<0.01 compared with untreated cells. **C** Time-dependent effects of I-BOP on MCP-1 protein expression. After 24 h serum-starvation, cells were treated with 50 nM I-BOP for the indicated time periods. Media were collected and proteins in each medium were concentrated by trichloroacetic acid (TCA) precipitation as described in Materials and methods. Concentrated samples were quantified by Bio-Rad protein assays to ensure equal protein loading for Western blot analysis. **D** Dose-dependent effects of I-BOP on MCP-1 protein expression in A549-TPα cells. **E** I-BOP-induced MCP-1 protein expression in A549-TPβ not A549-GFP cells. **F** I-BOP-induced MCP-1 protein expression in H157 and H460 cells. **G** Comparison of I-BOP with other prostanoids on the induction of MCP-1 in A549-TPα and H460 cells. Cells were treated with 50 nM I-BOP, 1 µM PGD_2_, 1 µM PGE_2_ or 1 µM PGF_2α_ for 24 h. The concentrations of prostanoids used here were optimized to induce the most expression of MCP-1. Media were collected and assayed as described in C. Densitometric analysis of each band was made, and control untreated time point is normalized to 1. All data are representative of at least three independent experiments.

To examine the effects of activation of TPα on the expression of MCP-1, A549-TPα cells were treated with TP agonist I-BOP (50 nM) at different time points. RT-PCR results showed that I-BOP induced a rapid and sustained expression of MCP-1 mRNA (Figure 1A). Quantitative real-time PCR assay was utilized to further determine the kinetics of MCP-1 induction. As shown in Figure 1B, the MCP-1 mRNA levels peaked at 4 h and still maintained a significant increase at 16 h after I-BOP treatment. The protein levels of I-BOP-induced MCP-1 expression were analyzed by Western blot assay. The accumulation of MCP-1 in conditioned medium reached the maximum level at 24 h following stimulation (Figure 1C). The induction of MCP-1 by I-BOP was dose-dependent and the effect was significant even at 1 nM of I-BOP (Figure 1D). In addition, I-BOP-induced MCP-1 expression can be observed in A549-TPβ but not in control A549 cells indicating both TPα and TPβ mediate MCP-1 expression (Figure 1E). Further, the induction of MCP-1 by I-BOP was also observed in H157 and H460 human lung cancer cells that express relatively high endogenous levels of TP [29] (Figure 1F). When compared with other prostanoids including PGD_2_, PGE_2_, and PGF_2α_, which were all used at their optimal concentrations, I-BOP induced the most significant expression of MCP-1 in A549-TPα and H460 cells (Figure 1G). These data show that TP plays an important role in the production of MCP-1 in lung cancer cells.

### SP1 is Important for TPα-mediated MCP-1 Expression

**Figure 2:**
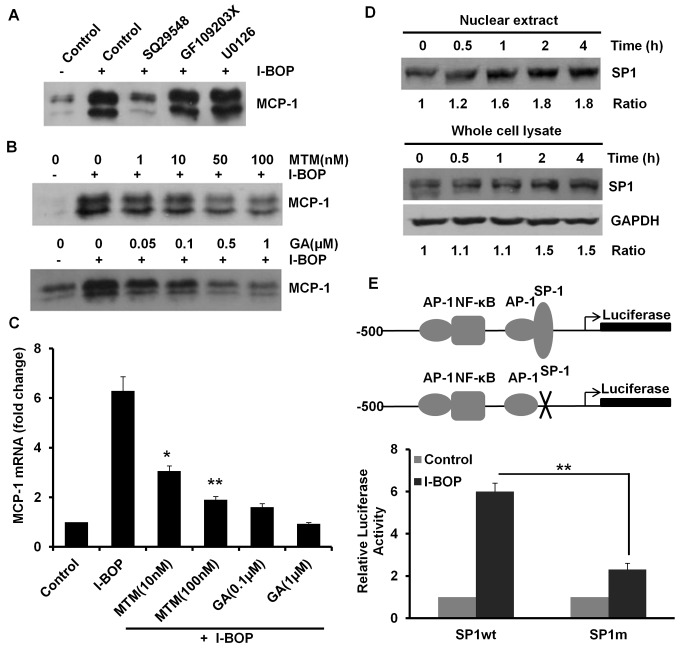
I-BOP regulates MCP-1 expression in SP1-dependent manner. **A** Effects of TP antagonist and several protein kinases inhibitors on MCP-1 induction by I-BOP stimulation. Cells were treated with TP antagonist SQ29548 (10 µM), PKC inhibitor GF109203X (0.5 µM), and MEK inhibitor U0126 (10 µM) for 30 min before incubation with 50 nM I-BOP for 24 h. Media were collected and assayed as described in Fig. 1C. **B** Effects of SP1 inhibition on I-BOP-induced MCP-1 expression at protein levels. Cells were treated with indicated concentrations of SP1 inhibitor mithramycin A (MTM) and Hsp90 inhibitor geldanamycin (GA) for 30 min before incubation with 50 nM I-BOP for 24 h. Media were collected and assayed as described in Fig. 1C. **C** Effects of SP1 inhibition on I-BOP-induced MCP-1 expression at mRNA levels. Cells were treated with above inhibitors for 30 min before incubation with 50 nM I-BOP for 4 h. RNAs were isolated and real-time PCR was performed as described in Materials and methods. *p<0.05 and **p<0.01 compared with I-BOP treated alone. **D** Protein level of SP1 was increased in the nuclei of A549-TPα cells following I-BOP stimulation. Cells were treated with 50 nM I-BOP for the indicated time periods. Nuclear extract and whole cell lysate were prepared as described in Material and methods. Protein level of SP1 was determined by Western blot. Densitometric analysis of each band was made and control untreated time point was normalized to 1. **E** Regulation of MCP-1 promoter activity by SP1. A549-TPα cells were transfected with luciferase reporter plasmids containing 500 bp of MCP-1 promoter sequence with either wild type or mutated SP1 binding sites. After 24 h transfection, cells were treated with vehicle control (0.1% ethanol) or 50 nM I-BOP for additional 18 h in serum free medium. Luciferase assay was carried out as described in Materials and methods. **P<0.01. All data are representative of at least three independent experiments.

Upon ligand binding, TP activates several downstream signal transduction cascades including PKC, and ERK pathways that are involved in cytokine-induced MCP-1 expression [15], [25], [30], [31]. To investigate whether these pathways are responsible for I-BOP-induced MCP-1 expression, several specific inhibitors were used. These inhibitors were used at concentrations proven to be optimal for each signaling molecule as described previously [25]. As shown in Figure 2A, except TP antagonist, SQ29548 at 10 µM, none of other inhibitors (0.5 µM GF109203X for PKC and 10 µM U0126 for MEK/ERK) has an effect on I-BOP-induced MCP-1 expression indicating these pathways are not involved in the induction. In the proximal region of MCP-1 promoter, there is a GC rich element, which can bind nuclear factor SP1 to activate transcription [12]. To elucidate whether SP1 is critical for MCP-1 induction by I-BOP, we employed mithramycin A (MTM), a specific SP1 inhibitor preventing SP1 from binding to its consensus GC rich sites [32], and geldanamycin (GA), an inhibitor of Hsp90 which was also reported to affect binding of SP1 to gene promoter [33]. Both inhibitors significantly suppressed the induction of MCP-1 by I-BOP at protein and mRNA levels (Figure 2B and C). We further investigated the effects of I-BOP on SP1 translocation and expression. As shown in Figure 2D, SP1 protein started to accumulate in the nucleus after incubation with I-BOP for 30 min, and remained elevated at 4 h. I-BOP also induced the expression of SP1 at 2 h. Moreover, promoter-luciferase reporter bearing a fragment of 500 bp of human MCP-1 gene promoter was used to evaluate the contribution of SP1 to I-BOP-induced MCP-1 transcription. SP1-binding element is located at −115 bp of MCP-1 promoter. Site-directed mutagenesis of SP-1 site resulted in a significant decrease of I-BOP-induced promoter activity compared with the wild type (Figure 2E). These data reveal that TP-mediated MCP-1 expression is SP1 dependent.

### I-BOP-induced MCP-1 Exhibits Chemotactic Effects on Macrophages

**Figure 3:**
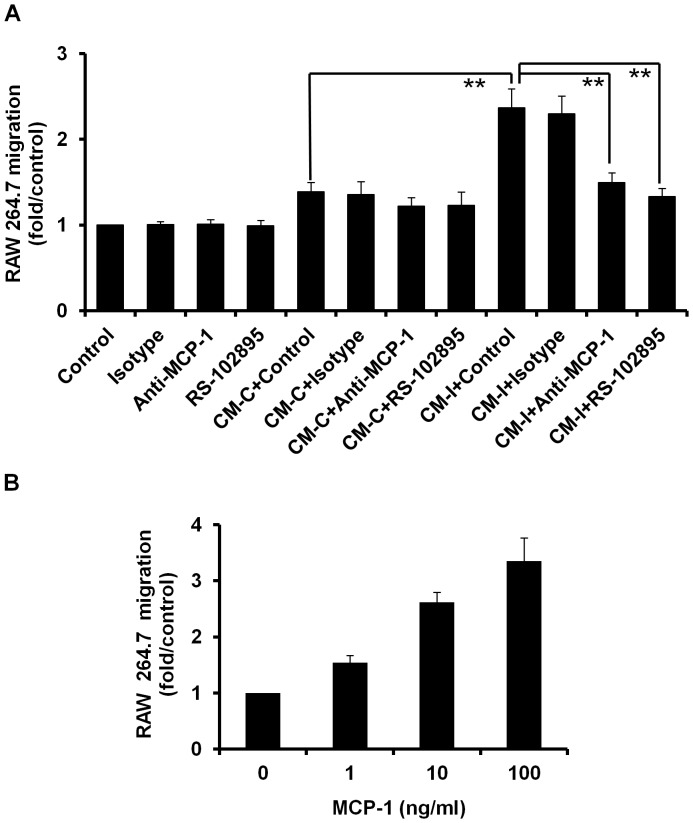
Chemotactic effects of A549-TPα conditioned media and MCP-1 on RAW 264.7 macrophages. **A.** Conditioned media increased the chemotaxis of RAW cells via MCP-1. CM-C is derived from vehicle-treated cells, and CM-I is derived from I-BOP-treated cells. SFM represents serum free medium. The concentrations of anti-MCP-1 antibodies and isotype control IgG were each at 5 µg/ml original CM. The concentration of RS-102895 was 10 µM. Detailed conditions were as described in Materials and Methods. **P<0.01. **B** Effect of recombinant MCP-1 on the chemotaxis of RAW cells. MCP-1 at 1, 10 and 100 ng/ml were tested as described in Materials and methods.

To determine the chemotactic property of I-BOP-induced MCP-1 on macrophages, we examined the effect of conditioned medium from A549-TPα cells treated with or without I-BOP on the migration of murine macrophage RAW 264.7 cells. As shown in Figure 3A, conditioned medium from I-BOP-treated cells significantly induced RAW 264.7 cell migration compared to the medium from vehicle (0.1% ethanol)-treated cells. MCP-1 neutralizing antibody and RS-102895, an antagonist of CCR2, significantly decreased the chemotactic potency of medium from I-BOP-treated A549-TPα cells suggesting that MCP-1 in the medium might be responsible for the effect. Indeed recombinant MCP-1 which was used as a positive control showed strong chemotactic effects on RAW 264.7 cells as expected (Figure 3B). These data indicate that MCP-1 is a key chemotactic factor in A549-TPα cell culture which caused migration of macrophages.

### Co-culture of RAW 264.7 Macrophages Induces Expression of Metastatic Genes by A549 Cells

**Figure 4:**
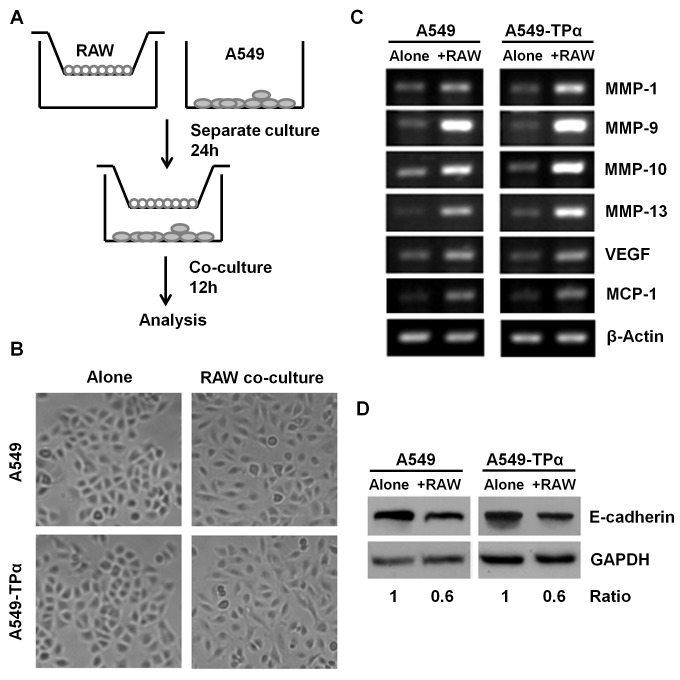
Transwell co-culture of RAW 264.7 macrophages induces metastatic gene expression by A549 cells. **A** A transwell co-culture system of RAW 264.7 macrophages and A549 cells was used in this study. RAW 264.7 cells and A549 cells were separately cultured for 24 h and then co-cultured in serum free medium for further 12 h or 24 h as described in Materials and methods. **B** Control A549 and A549-TPα cells became more scattered and spindle shaped after culture with RAW 264.7 cells. Both types of A549 cells were pictured after 12 h of culture with or without RAW 264.7 cells as described in A. **C** Co-culture of RAW 264.7 macrophages induced metastatic gene expression by A549 cells. After 12 h of co-culture with macrophages, the expression of several metastatic genes in control A549 and A549-TPα cells, including MMPs, VEGF and MCP-1 mRNA, were examined by RT-PCR as described in Materials and methods. **D** Co-culture of RAW 264.7 macrophages decreased E-cadherin expression by A549 cells. Control A549 and A549-TPα cells were collected after 24 h of culture with or without RAW 264.7 cells as described in A. Protein level of E-cadherin was determined by Western blot as described in Material and methods. All data are representative of at least three independent experiments.

To elucidate the effects of macrophages on the invasiveness of A549 cells, we first used a trans-well co-culture system as shown in Figure 4A. In this co-culture system, there is no cell-cell direct contact and cells communicate with each other through soluble proteins. Both control A549 and A549-TPα cells became more elongated and scattered after 12 h of co-culture with RAW 264.7 cells than the cells grown alone (Figure 4B). Furthermore, results from RT-PCR analysis showed that expression of metastatic genes including several *MMPs* and *VEGF* were significantly elevated in either control A549 or A549-TPα cells co-cultured with macrophages (Figure 4C). Macrophages also induced *MCP-1* gene expression by control A549 or A549-TPα cells (Figure 4C). E-cadherin-catenin complex is critical for cell adhesiveness and maintenance of normal tissue architecture. Reduction of E-cadherin is tightly linked with cell migration and invasion [34], [35]. Therefore, we examined whether macrophage-lung cancer cell interactions regulate E-cadherin expression in control A549 or A549-TPα cells. Indeed, co-culture with RAW 264.7 macrophages significantly reduced the levels of E-cadherin in either type of A549 cells (Figure 4D). All of these results were reproducible in wild type A549 cells without GFP or TPα expression (data not shown). Collectively, these data indicate that co-culture with macrophages may increase migration and invasion potential of A549 cells.

### Co-culture of RAW 264.7 Macrophages Induces Morphological Change and Invasion of A549 Cells

**Figure 5:**
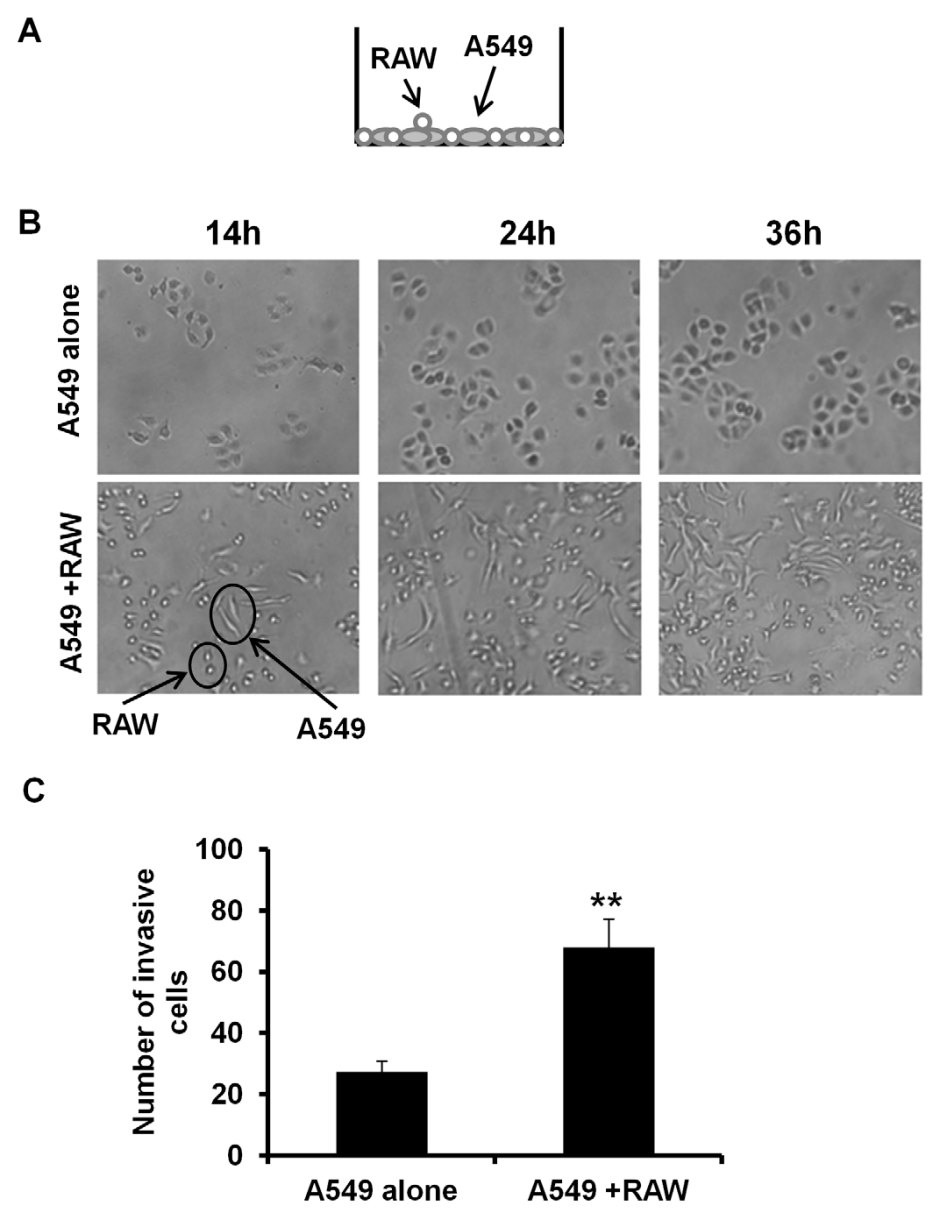
Direct co-culture of RAW 264.7 macrophages induces morphological change and invasion of A549 cells. **A.** A direct co-culture system of RAW 264.7 macrophages and A549 cells was used in this study. RAW 264.7 cells and A549 cells were plated into the same well of a culture plate. **B.** A549 cells exhibit elongated shape after culture with RAW 264.7 macrophages. The control A549 cells were plated into a 12-well plate with or without RAW 264.7 cells as described in A. Cells were pictured at the indicated time points. In the lower panel, those small cells exhibiting round shape were RAW 264.7 cells. **C.** Co-culture of RAW 264.7 macrophages induced invasion of A549 cells. Invasion assay of A549 cells was carried out as described in Material and methods. **P<0.01. Results are representative of three independent experiments.

To further examine if macrophages promote invasion of A549 cells, we next used another co-culture system as shown in Figure 5A. In this system, cells communicate with each other via direct contact, which is closer to the physiological situation. As shown in Figure 5B, after 12 h of co-culture with macrophages, control A549 cells exhibit elongated protrusions indicating the invasion potential. This morphology still sustained after 36 h of co-culture. In addition, in matrigel invasion assay, co-culture with macrophages induced a 2.5-fold increase in cellular invasiveness of control A549 cells through matrigel relative to those cells grown alone (Figure 5C). These results indicate that macrophages attracted by MCP-1 released from A549 cells may stimulate cancer cells invasion.

## Discussion

The relationship between over-expression of TXAS or TP, two necessary components for TXA_2_ production and function, and cancer invasion and metastasis was observed in a wide range of cancers including NSCLC [36], [37]. However, there are few studies revealing the downstream targets by which TXA_2_-TP signaling axis mediates these processes. In the present study, we identified chemokine MCP-1 as an important product of TP signaling in lung cancer A549 cells expressing TPα ectopically. Our findings suggest that MCP-1 may attract macrophages to cancer cells, thereby promoting their invasion.

TXA_2_ has been shown to induce MCP-1 expression in vascular endothelial cells and also other cytokines in WISH cells (an amnion epithelium-derived cell line) [18], . In addition, a recent report showed that TXAS inhibitor ozagrel suppressed MCP-1 and IL-8 gene expression and inflammatory cell accumulation in a lung injury model indicating the role of TXA_2_ in regulation of these processes [39]. Cancers and chronic inflammation display significant parallels. Therefore, they share certain characteristics. Several CC chemokines including MCP-1 were found to be highly expressed in human NSCLC tumor tissues and the levels of MCP-1 also significantly correlated to macrophage infiltration [9]. These findings have prompted us to hypothesize that TXA_2_ might be involved in lung cancer development via MCP-1-mediated macrophage recruitment.

Mechanisms of regulation of MCP-1 by I-BOP were further studied. There are several cis-acting transcriptional regulatory elements including AP1 and SP1 sites in the promoter region of human *MCP-1* gene. Activation of AP-1 by TP-mediated G protein signaling has been found in several other cell types including A549 cells [25]. We previously found that I-BOP-induced MMP-1 expression was mediated by PKC and ERK-dependent AP-1 activation in the same cell lines [24]. Nevertheless, inhibition of ERK and PKC did not block I-BOP-induced MCP-1 expression, suggesting AP-1 was not involved in MCP-1 regulation by I-BOP in A549-TPα cells. These data indicated that separate signaling pathways were involved in the expression of downstream targets induced by I-BOP activation of TP. It is also possible that MCP-1 regulation is tissue-specific. AP-1 is not important for MCP-1 expression in lung epithelial cancer cells. A separate study in fibroblast 3T3 cells showing that the expression of MMP-1 induced by growth factors was c-Fos-dependent, whereas that of MCP-1 was not provides support to our proposition [40]. Moreover, it has been reported that PDGF induction of MCP-1 transcription requires SP1 but not AP-1 response elements in its promoter region in fibroblast [12]. Therefore, we propose that in lung cancer cells I-BOP may regulate MCP-1 expression in a way similar to how PDGF does in fibroblast. We observed that SP1 was increased in the nucleus after I-BOP stimulation and SP1 inhibitor blocked I-BOP-induced MCP-1 expression. Furthermore, using GC-rich promoter region of 12(S)-lipoxygenase as a probe, Chang and his colleagues identified that Hsp90 interacts with SP1 and modulates its promoter binding ability [33]. In the present study, indirect inhibition of SP1 by Hsp90 inhibitor geldanamycin blocked I-BOP-induced MCP-1 expression at protein as well as mRNA levels. Moreover, the results obtained from MCP-1 promoter-luciferase reporter assay further indicate the critical role of SP1 binding site in MCP-1 regulation by I-BOP. Although the upstream signals by which TP regulates SP1 expression and translocation need further studies, the results presented here demonstrated that I-BOP induced MCP-1 expression in A549-TPα cells by SP1-dependent but AP-1-independent mechanism.

It has been shown that the increased accumulation of macrophages in the tumor microenvironment was associated with poor prognosis of several types of cancers such as prostate, breast, bladder, and esophageal cancers [41]–[44]. For lung cancer, macrophages as prognostic factor is still debated since the data obtained from clinical studies seem controversial [9], [45], [46]. This may be due to the infiltration of different types of macrophages. Macrophages have been classified into two major groups: (a) M1 macrophages possess pro-inflammatory properties and (b) M2 macrophages have anti-inflammatory phenotype and promote tumor progression. Within the tumor islet, both M1 and M2 macrophages could be present. Those macrophages associated with favorable outcome in NSCLC patients were identified as M1 macrophages [47]. M2 macrophages may function in an opposite way. Zeni *et al.* [48] reported that the percentage of IL-10-positive macrophages within tumor islet was higher in patients with late stage of NSCLC having lymph node metastasis than those patients with early stage of NSCLC having no metastasis, and these macrophages also predicted the worse overall survival. High expression of IL-10 is one of the characteristic of M2 phenotype [49], thus this study suggested that these macrophages behaved as M2-polarized TAMs and thereafter promoted progression of NSCLC.

We employed RAW 264.7 macrophages to study the interactions between lung cancer cells and macrophages. RAW 264.7, a murine macrophage cell line, can be polarized into either M1 or M2 macrophages [50]. Interactions of RAW 264.7 macrophages with human cancer cells such as lymphoma and breast cancer cells have been reported in previous studies [51], [52]. Furthermore, human MCP-1 and mouse MCP-1 truncated at the C terminus are highly homologous. Indeed, human MCP-1 even has a higher chemotactic potency to murine monocytes than full length of murine MCP-1 due to the less glycosylation of human MCP-1 C terminus [53]. Therefore, RAW 264.7 macrophages can serve as an appropriate cell line to study TP-mediated interactions between lung cancer cells and macrophages.

Macrophages contribute to various aspects of cancer progression such as cancer cells proliferation, survival, invasion, metastasis and angiogenesis. In this study, co-culture of macrophages with A549 cells stimulated gene expressions of several MMPs and VEGF by A549 cells. We previously reported that A549-TPα cells induced tumor formation and angiogenesis in nude mice through induction of VEGF expression [54]. We recently found that I-BOP-induced invasion of A549-TPα cells was mediated by increased expression of several MMPs such as MMP-1 and MMP-9 [24]. Here, macrophages induced these genes, and thus enhanced the effects of TP-mediated angiogenesis and invasion of cancer cells. MCP-1 was also induced in A549 cells by co-culture with macrophages forming a positive feedback loop that might reinforce the recruitment of more macrophages to promote cancer cell invasion. We did not examine the factors released by RAW cells that may induce the expression of metastatic genes. However, there is one report showing that breast cancer cells stimulated production of inducible nitric oxide synthase and nitric oxide in RAW 264.7 macrophages, which in turn induced *MMP9* and *VEGF-A* gene expression in breast cancer cells [52]. This is also likely the mechanism by which RAW cells stimulated expression of metastatic genes in lung cancer cells.

In addition to its indirect effects on tumor progression by recruiting macrophages, MCP-1 has been shown to act directly on cancer cells, such as prostate cancer, colon cancer and bladder cancer cells to stimulate their migration and invasion [55]–[57]. In this report we focused on the indirect effects of MCP-1 in recruiting macrophages to enable invasion of lung cancer cells. Whether macrophages can be induced to express MCP-1 acting directly on lung cancer cell invasion remains to be determined. Further studies on the induction of MCP-1 in macrophages and on the direct effects of MCP-1 on the invasion of lung cancer cells will be the focuses of our future reports.

**Figure 6:**
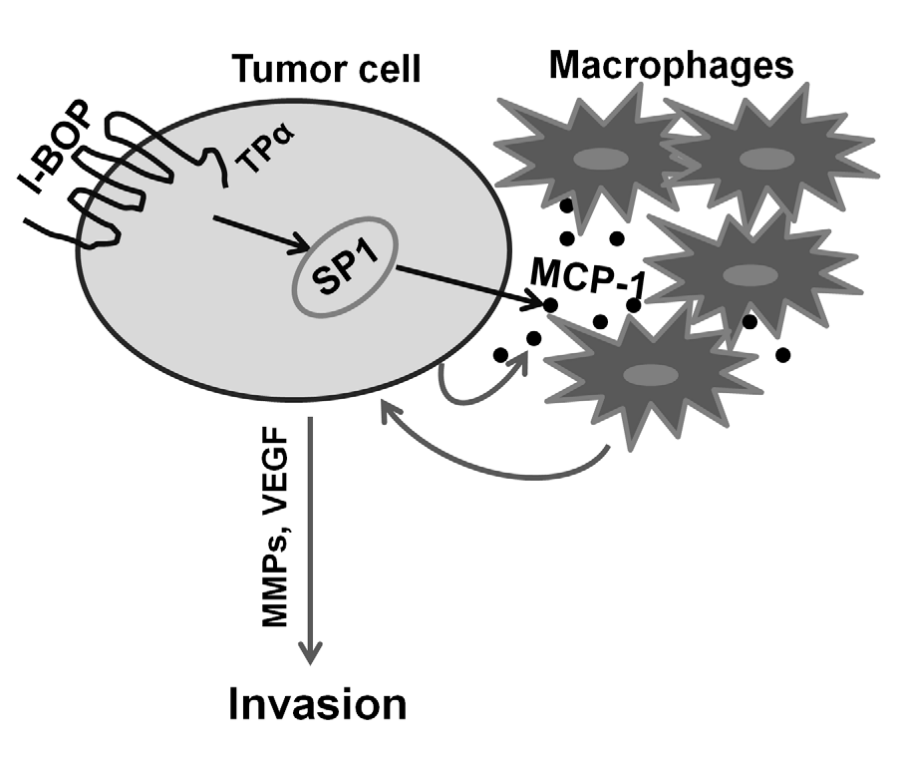
Proposed scheme of TP-mediated interactions between cancer cells and macrophages.

Tumors are very heterogeneous and contain numerous subpopulations of cells [58] that may express different levels of TP. We show here that once TXA_2_ initiates a signaling cascade leading to the release of MCP-1 from tumor cells expressing high levels of TP, the subsequent recruited macrophages could increase the invasion of tumor cells regardless of their TP levels. Therefore, TP expands its influence on tumor progression through MCP-1-mediated macrophage recruitment. In summary, TP-mediated interactions between cancer cells and macrophages leading to an increased invasive potential of carcinoma cells is illustrated in Figure 6.
